# Mental Rotation Test Performance in Brazilian and German Adolescents: The Role of Sex, Processing Speed, and Physical Activity in Two Different Cultures

**DOI:** 10.3389/fpsyg.2019.00945

**Published:** 2019-04-26

**Authors:** Petra Jansen, Flávia Paes, Sabine Hoja, Sergio Machado

**Affiliations:** ^1^ Faculty of Psychology, Pedagogic and Sport Science, University of Regensburg, Regensburg, Germany; ^2^ Institute of Psychiatry, Federal University of Rio de Janeiro, Rio de Janeiro, Brazil; ^3^ Laboratory of Physical Activity Neuroscience, Physical Activity Science Postgraduate Program, Salgado de Oliveira University, Niterói, Brazil

**Keywords:** mental rotation, adolescents, physical activity, Brazil, Germany, cross cultural research

## Abstract

It was the main goal of this study to investigate performance on the mental rotation test (MRT) in Brazilian and German adolescents. Mental rotation is the ability to mentally transform a three-dimensional stimulus in mind and relates to science education. 60 German and 60 Brazilian adolescents (76 males and 44 females, 11–17 years) completed the Mental Rotation Test, a physical activity and media use questionnaire and a Number Connection Test. The result showed no difference between Brazilian and German adolescents in the cognitive processing speed measurement. German adolescents are more active and show a less media use compared to the Brazilian adolescents. Furthermore, German adolescents demonstrate a better MRT performance than Brazilian ones, as well as boys show a better performance than girls do. A multiple regression analysis indicated that the MRT performance could be predicted by nationality, sex, and cognitive processing speed. Since cognitive processing speed did not differ between Brazilian and German adolescents, the worse MRT performance of the Brazilian adolescents could be explained by different educational systems. Further studies have to follow which investigate the reasons for the different nations in more detail.

## Introduction

Mental rotation is defined as the ability of mental transform of how an object appears if it is rotated from its original position (Shepard and Metzler, 1971). This specific spatial task is well examined in different fields, such as sex differences (Jansen-Osmann and Heil, 2007), developmental psychology (Quinn and Liben, 2008), neuroscience (Jordan et al., 2001), and general psychology (Yuille and Steiger, 1982; Bethell-Fox and Shepard, 1988).

Differences in spatial abilities between males and females favoring males are clearly established findings, with MRT performance producing the largest effects (Voyer et al., 1995) and being significantly larger with time constraints (Voyer, 2011). Also the use of different strategies (Hirnstein et al., 2009) and the confidence in the judgment (Estes and Felker, 2012) influences possible sex differences. However, Jansen-Osmann and Heil (2007) investigated sex^1^ differences in chronometric tasks with different types of stimuli (see also Iachini et al., 2019) and a better male performance could only be shown for polygon stimulus.

Studies that investigated the effects of long-term physical activity on MRT performance found better MRT performance for students of sports sciences compared to students of educational sciences (Pietsch and Jansen, 2012). Also motor experts have a better spatial ability (Voyer and Jansen, 2017). At this point, it is unclear, if this motor expert effect is also evident in people who are only more physical active than others.

There are only a few studies investigating cultural differences regarding Mental Rotation Test performance: Omani students show a worse MRT performance compared to German students (Jansen et al., 2016), and children in Cameroon have lower scores than children in Germany (Jansen et al., 2018). Contrary to the prediction of the social role theory that sex differences are larger in sex non-egalitarian nations, it has been shown that the sex difference in MRT performance is higher in egalitarian nations (Lippa et al., 2010). According to the Gender Gap Report 2018 (World Economic Forum, 2018), Brazil’s rank is 95 and the rank of Germany 14 out of 149 countries with higher rankings indicating a larger sex gap and therefore a smaller sex difference in MRT performance (Lippa et al., 2010).

With regard to cross-cultural cognitive differences between German and Brazilian children, German children scored higher than Brazilian children in intellectual and emotional autonomy in a self-rated survey by school teachers and students (Schwartz, 1994), and the German educational system is rated be better than the Brazilian one (Lugtmeijer, 2017). A comparison in the spatial abilities and motor behavior between both countries is missing.

Thus, the objective of this study is to investigate whether there are performance differences in solving the MRT between Brazilian and German boys and girls and the contributing factors of cognitive processing speed, physical activity, and media usage. According to the literature presented above, we hypothesize (1) that the MRT performance should be better in German than Brazilian adolescents and (2) that the sex differences should be smaller in the Brazilian than German sample. Furthermore, we explore the possible role of nationality, age, sex, cognitive processing speed, sports practice, and media usage in predicting the MRT performance.

## Materials and Methods

### Participants

Around 120 children aged between 11 and 17 years (mean age *M* = 13.42 years, *SD* = 1.80), half German (38 males, *M* = 13.61 years, *SD* = 1.82; 22 females, *M* = 13.09 years, *SD* = 1.74), half Brazilian (38 males, *M* = 13.61 years, *SD* = 1.82; 22 females, *M* = 13.09 years, *SD* = 1.74) participated in the current study. Regarding age and sex, the Brazilian children were matched to the German sample.

Parents of the participating children gave written informed consent prior to the study and the data were collected and analyzed anonymously. The study was approved as part of a larger project by the Human Research Ethics Committee of the Salgado de Oliveira University (38227014.0.0000.5289). The study was conducted according to the guidelines of the declaration of Helsinki.

### Material

#### Demographic Questionnaire

Demographic data of the participants concerning sex, age, and time spent practicing sport were recorded with a self-generated questionnaire. Hours of practice and use of electronic devices were registered by the questionnaire used in the health behavior in school-aged children (HBSC) study protocol (Currie et al., 2010) including the subscales physical activity. For analysis of physical activity, the item “How many hours a week do you usually exercise in your free time so much that you get out of breath or sweat?” with a response scale from “1 = none” to “6 = about 6 h or more” was used.

#### Number Connection Test

The number connection test (ZVT) (Oswald, 2016) was administered to measure cognitive processing speed. The test consists of four sheets in which each of these pages contains numbers from 1 to 90 in a different order. The numbers have to be connected in ascending order with a pencil as fast as possible. 30 s were given to solve each page. The number of correctly marked numbers within 30 s was transformed in ZVT scores. The internal consistency and the 6-month test-retest reliability of the ZVT are about 0.90–0.95.

#### Mental Rotation Test

The mental rotation test (MRT-A) test is a speeded mental rotation test and appropriate to use for children and adolescents older than 10 years of age (Peters et al., 1995; Hoyek et al., 2012). The test consists of 24 items, divided into two sets of 12 tasks each. Every item is comprised of an initial cube structure and four possible rotated cubes. The task is to choose the two cube figures that can be converted back into the initial by mental rotation in a period of 3 min per set with a 30-s break in between. A task is solved correctly if both answers were identified and one point was given. One item explained by the investigator and three training items were included before the 24 test items. The accuracy score can vary between 0 and 24.

### Procedure

The pupils were tested in each country in group sessions, in the following order: (1) demographic questionnaire; (2) physical activity and sedentary behavior questionnaires; (3) cognitive processing speed test; (4) mental rotation test.

### Statistics

To investigate if there are differences in the physical activity (hours per week) and media behavior (hours per week), two analyses of variance with the dependent variables (1) “hours of sports practice per week” and (2) “hours of media behavior per week” were conducted, with the independent factors “nationality” (German and Brazilian) and “sex” (boys, girls). For the media behavior, the use of electronic devices, TV, video and computer games, chatting, emailing etc., were meant.

Two univariate analyses of variance were conducted for the dependent variables (1) “cognitive processing speed,” and (2) “number of correctly solved items in the MRT,” with the independent factors “nationality” (German and Brazilian) and “sex” (boys, girls). Furthermore, a multiple regression on MRT performance with the predictors’ nationality, age, sex, cognitive processing speed, hours of sports practice per week, and hours of media used per week was conducted to investigate the relevant predictors for MRT performance.

## Results

### Physical Activity and Media Behavior

The analysis regarding physical activity revealed a significant main effect for the factor “nationality,” *F*(1, 116) = 38.92, *p* < 0.001, partial *η*^2^ = 0.25 (German *M* = 4.10, *SD =* 1.19, Brazilian: *M* = 2.65, *SD* = 1.16), as well as in use of electronic devices likewise for “nationality” *F*(1, 116) = 33.22, *p* < 0.001, partial *η*^2^ = 0.22 (German *M* = 2.89, *SD* = 1.0, Brazilian *M* = 3.72, *SD* = 0.43). Both analyses revealed neither an effect for sex, physical activity: *F*(1, 116) = 2.92, *p* = 0.09, partial *η*^2^ = 0.02, media behavior: *F*(1, 116) = 0.39, *p* = 0.53, partial *η*^2^ < 0.01, nor an interaction for “nationality” and “sex,” physical activity *F*(1, 116) = 1.65, *p* = 0.2, partial *η*^2^ = 0.01; media behavior: *F*(1, 116) = 0.57, *p* = 0.45, partial *η*^2^ < 0.01.

### Number Connection Test

A univariate analysis of variance with the dependent variable “cognitive processing speed” showed no significant differences for the factor “sex,” *F*(1, 116) = 1.085, *p* = 0.300, partial *η*^2^ = 0.009 nor for the factor “nationality,” *F*(1, 116) = 0.73, *p* = 0.394, partial *η*^2^ < 0.006 and neither a significant interaction between both factors, *F*(1, 116) = 0.17, *p* = 0.681, partial *η*^2^ = 0.001. The ZVT values for the German girls (*M* = 108.45, *SD* = 7.87) and boys (*M* = 106.37, *SD* = 7.47) did not differ. There was also no difference between the Brazilian girls (*M* = 106.64, *SD* = 0.70) and boys (*M* = 105.74, *SD* = 7.79).

### Mental Rotation Test

#### Number of Correctly Solved Items

**Figure 1:**
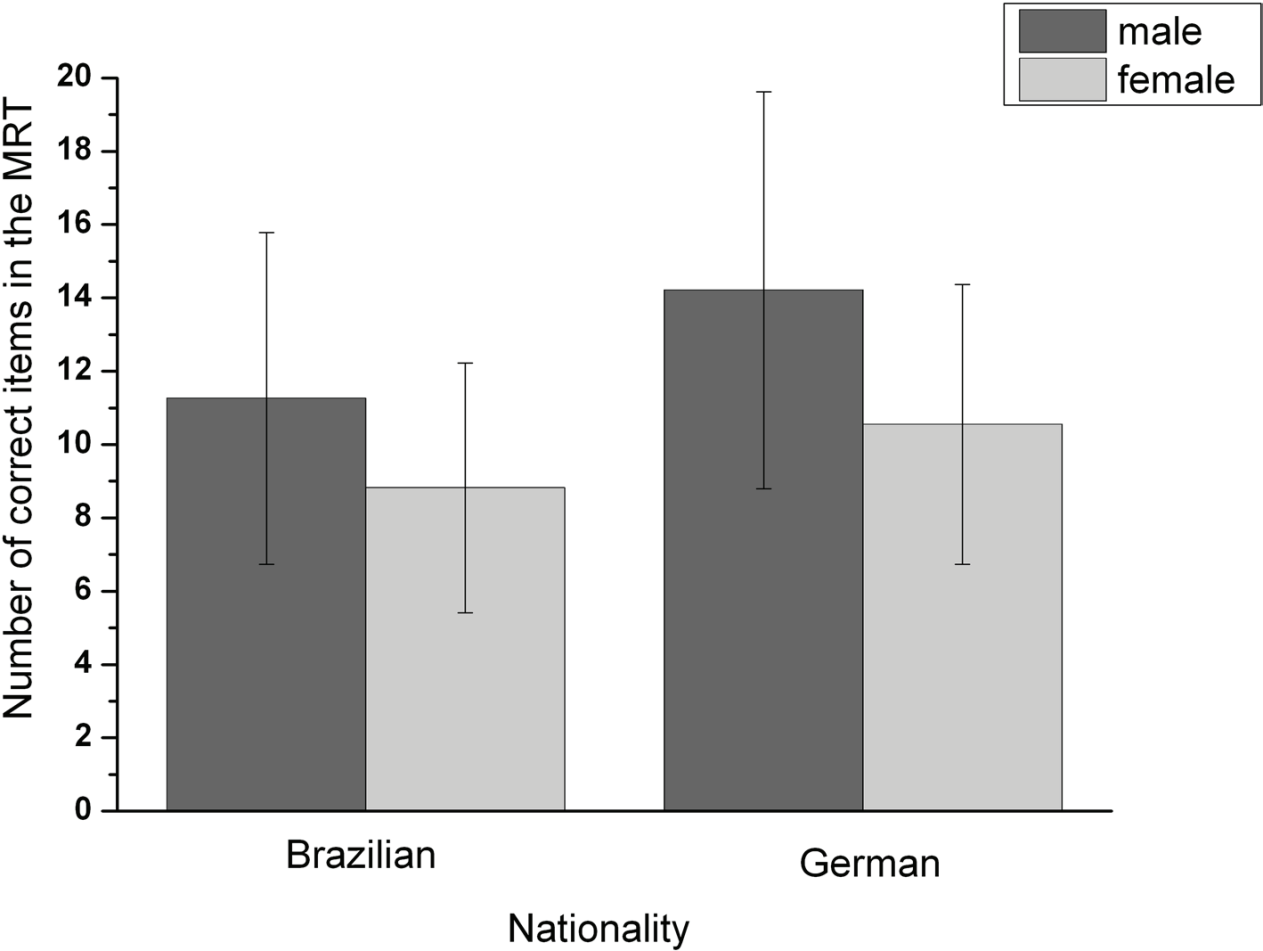
Means and standard deviation of the number of correct items in the MRT dependent on nationality and sex.

In the analysis of the standard performance measurement with the MRT, our results show that there was a significant effect of the factor “nationality,” *F*(1, 116) = 7.38, *p* = 0.008, partial *η*^2^ = 0.06. Adolescents from Brazil (*M* = 10.37, *SD* = 4.28) performed significantly worse than the ones from Germany (*M* = 12.87, *SD* = 5.17). There was also a significant effect of the factor “sex,” *F*(1, 116) = 12.61, *p* = 0.001, partial *η*^2^ = 0.01, but no significant interaction between “nationality” and “sex,” *F*(1, 116) = 0.503, *p* = 0.480, partial *η*^2^ = 0.004. Boys (*M* = 12.74, *SD* = 5.17) showed a better performance than girls (*M* = 9.68, *SD* = 3.67), see Figure 1.

### Regression

The multiple regression with including all variables results on MRT performance with the predictors nationality, age, sex, cognitive processing speed, hours of sports practice per week, and hours of media used per week showed that 23.8% of the variance (corrected *R^2^* = 19.7%) was explained by the predictors “cognitive processing speed,” “nationality,” and “sex,” *F*(6, 113) = 5.951, *p* < 0.001, see Table 1. A stepwise regression analysis leads to the same results.

**Table 1:** Multiple regression results for the criterion mental rotation test performance with the predictors nationality, age, sex, cognitive processing speed, hours of sports practice per week and hours of media.

Predictor	*β*	*T*	*p*
Nationality	−0.368	−3.494	0.001
Sex	−0.336	−3.980	0.000
Age	0.058	0.667	0.506
Cognitive processing speed	0.243	2.940	0.004
Physical activity	−0.129	−1.288	0.200
Media time	0.097	1.016	0.312

## Discussion

The results of the study demonstrate that Brazilian adolescents show a worse MRT performance compared to German ones, boys are better than girls and physical activity does not contribute to mental rotation test performance.

The worse MRT performance of Brazilian adolescents is in line with our first hypothesis. One reason for this might be the different scholastic quality in both countries (Lugtmeijer, 2017). This assumption is in line with a study of Titze et al. demonstrating that scholastic systems (co-educative schools vs. same sex schools) influence MRT scores (Titze et al., 2011). Moreover, the pressure of group testing might be different in both countries and so the asymmetry in the competitiveness of the testing environment can influence the results.

Because we did not find an interaction between the factors “nationality” and “sex,” our second hypothesis could not be confirmed. The result is in contrast to the results of the study of Lippa et al. (2010) but also to the social role theory. Next to social explanations, biological theories might play a relevant role. The biological approach addresses the levels of sexual hormones (Courvoisier et al., 2013), the cerebral lateralization (Hahn et al., 2010) and the level of prenatal hormones (Heil et al., 2011). But to conclude, the different MRT performance between males and females is far away from being understood in detail.

With regard to our third goal of the paper, it can be stated that an average amount of physical activity is not sufficient to reach a better spatial competence. The motor expert effect (Voyer and Jansen, 2017) in MRT performance seemed to be more relevant for people who are motor experts in one specific motor domain contrary to people who are just more active. The result is contradictory to the relation of executive functions and inhibition, for example, where it has been shown that fit children show a better inhibition performance then children who are less fit (Hillman et al., 2009).

## Conclusion

This study provides insight in spatial cognitive development in Brazilian and German female and male adolescents.

## Ethics Statement

Parents of the participating children gave written informed consent prior to the study and the data were collected and analyzed anonymously. The study was approved as part of a larger project by the Human Research Ethics Committee of the Salgado de Oliveira University (38227014.0.0000.5289). According to this, the quasi-experimental study was conducted according to the guidelines of the declaration of Helsinki.

## Author Contributions

PJ designed the study, analyzed the data, and wrote the first draft of the paper. SH helped to design the study, organized the data acquisition, and discussed the first draft of the paper. FP and SM acquired the data and discussed the first draft of the paper.

### Conflict of Interest Statement

The authors declare that the research was conducted in the absence of any commercial or financial relationships that could be construed as a potential conflict of interest.

## Footnotes

^1^The use of the term “sex” is based upon genetic classification and not the participant’s gender identity.
